# Close to community health providers post 2015: Realising their role in responsive health systems and addressing gendered social determinants of health

**DOI:** 10.1186/1753-6561-9-S10-S8

**Published:** 2015-12-18

**Authors:** Sally Theobald, Eleanor MacPherson, Rosalind McCollum, Rachel Tolhurst

**Affiliations:** 1 International Public Health Department, Liverpool School of Tropical Medicine, Pembroke Place, Liverpool, L3 5QA, UK

## Abstract

Universal health coverage is gaining momentum and is likely to form a core part of the post Millennium Development Goal (MDG) agenda and be linked to social determinants of health, including gender;

Close to community health providers are arguably key players in meeting the goal of universal health coverage through extending and delivering health services to poor and marginalised groups;

Close to community health providers are embedded in communities and may therefore be strategically placed to understand intra household gender and power dynamics and how social determinants shape health and well-being. However, the opportunities to develop critical awareness and to translate this knowledge into health system and multi-sectoral action are poorly understood;

Enabling close to community health providers to realise their potential requires health systems support and human resource management at multiple levels.

## Introduction

There has been a growing commitment to the goal of universal health coverage (UHC) and wide reaching and high level discussion about the centrality of equity and UHC in the discussions preceding the Sustainable Development Goal (SDG) with SDG 3 aspiring to ‘Ensure healthy lives and promote well-being for all at all ages’ and target 3.8 aiming to ‘Achieve universal health coverage, including financial risk protection, access to quality essential health-care services and access to safe, effective quality and affordable essential medicines and vaccines for all’ .. The ‘United Nations (UN) conference on Sustainable Development (Rio+20) declaration states that: “We … recognize the importance of universal health coverage to enhancing health, social cohesion and sustainable human and economic development. We pledge to strengthen health systems towards the provision of equitable universal coverage. We call for the involvement of all relevant actors for coordinated multi-sectoral action to address urgently the health needs of the world's population.”[1,2].

Understanding health as part of sustainable development requires a shift of emphasis of health sector development towards addressing the social determinants of health, including gender[3]. Meeting the goal of universal health coverage and supporting social development will require action and dedication of resources at all levels and building blocks within the health system. This will necessarily include joint multi-sectoral collaboration beyond the health system.

Over the forthcoming 10-20 year horizon, an epidemiological transition towards an increasing chronic and non-communicable disease (NCD) burden in the global South is expected. This will require significant re-shaping of health service provision models to enable the long-term treatment and management of chronic health conditions and the re-orientation of public health strategies towards non-communicable disease prevention, including multi-level and multi-institutional collaboration[4]. The very nature of chronic diseases, requiring long term services and multiple contacts are likely to intensify demands on both the health system and close-to-community (CTC) providers for treatment and prevention.

The health workforce is a key health systems building block that underpins the expansion of health services. Most countries in the global South have a shortage of formal health workers and are increasingly looking to a range of CTC providers to fill the gap, and in particular reach the poorest and most marginalised individuals, households and communities. There are many types of CTC providers, including but not limited to community health workers (CHWs), village midwives, traditional birth attendants (TBAs), formal and informal private practitioners (IPPs), community based drug distributors (CDDs) and lay counsellors, all of whom deliver a wide range of services in different contexts.

In the past decade, there has been a growing recognition of the contribution and potential of CTC providers and particularly CHWs as an integral component of the health workforce needed to achieve the MDGs[5]. The increasing focus on achieving universal coverage has led to a revitalisation of CHW programmes; with some countries implementing health system extensions using CHWs nationwide and others considering options for scale-up. CHWs' current roles typically include education; health promotion, immunisation; management of disease outbreaks, community mobilization; counselling; screening and point-of-care diagnostics; follow-up and referral; data collection, community based drug distribution and basic treatment and care for some diseases. Their scope of work ranges from maternal and child health, including nutrition, to sexual and reproductive health, HIV, malaria and TB diagnosis.

CTC providers are sometimes a formally employed cadre who are remunerated and integral to the health system; but they can also be volunteers who are brought on board for ad-hoc activities (for example in the case of community-based/directed drug distributors for NTDs) without formal contractual arrangements with the system. It is estimated that most (70% globally) CHWs are female[6]. In some contexts they are all female by policy (e.g. in Ethiopia). CTC health programmes often rely on staff who live and work at the community level, and engage with local residents in their dwellings or workplace. Their location generates potential to strengthen delivery of health services to meet the specific needs and realities of individuals and households, linking the community with the formal health system and beyond.

This paper aims to identify some key areas where there are opportunities for CTC providers to support health systems in strengthening universal health coverage, and to consider their potential from social determinants of health and gender equity perspectives. The paper covers three themes. Firstly it discusses the role of CTC providers as extenders of services, secondly it considers their potential as social change agents and thirdly it briefly reviews the need to manage CTC providers to support them in carrying out their roles. We draw on existing evidence and make particular reference to NTDs, maternal and newborn health and lung health (as core areas for the CAHRD consultation) and identify key areas for research and action.

**Figure 1:**
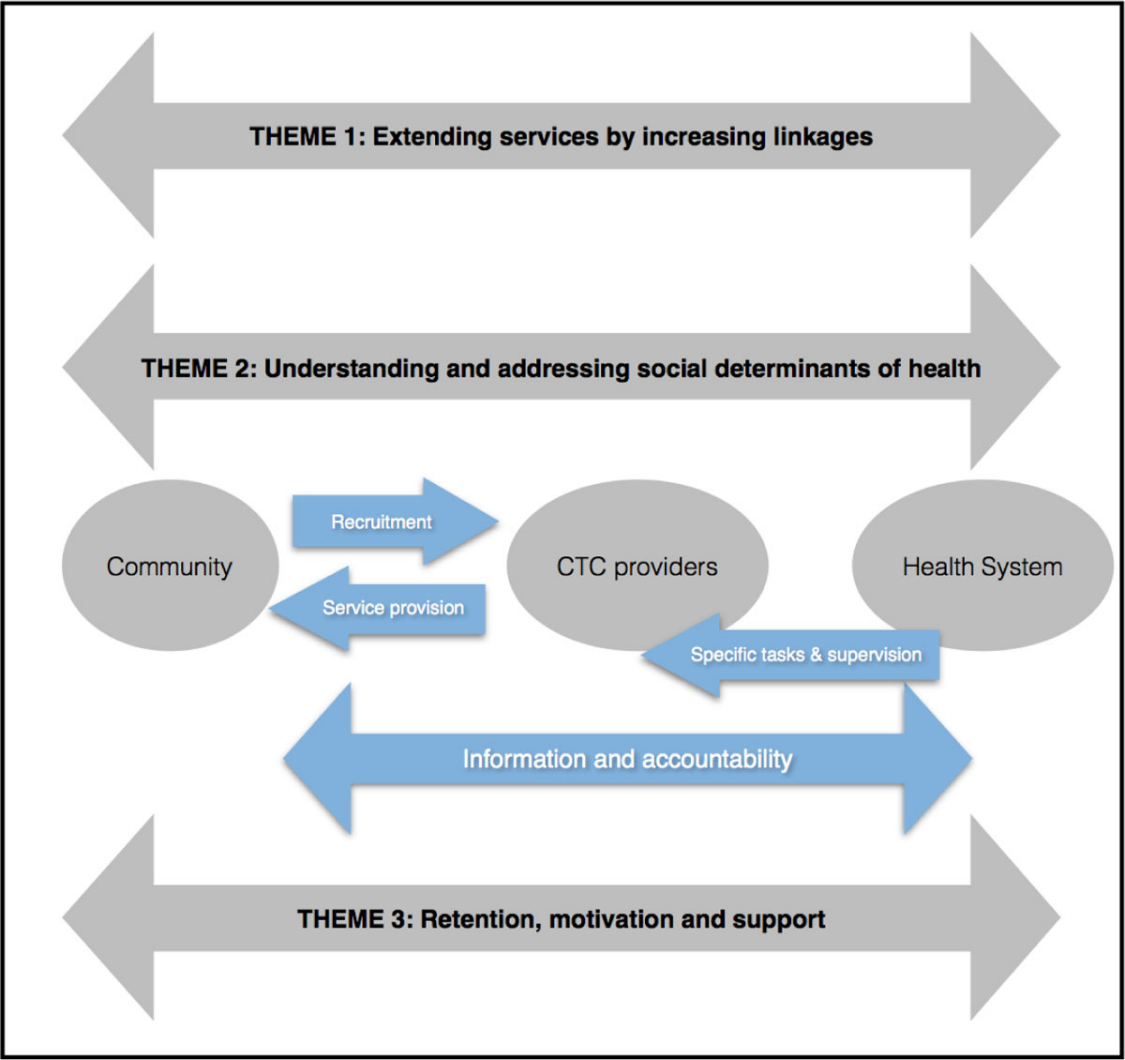
Conceptual map for the paper

Figure 1 lays out the conceptual basis for the paper illustrating the three key themes and the relationships between and activities linking communities, CTC providers and health systems.

## 1. CTC providers as service extenders: UHC with a focus on vulnerable and marginalised individuals and communities

CTC service providers and in particular CHWs are often introduced as part of strategies to extend primary health services to underserved communities at low cost in contexts of chronic financial and human resource shortages[7,8]. Whilst this may extend coverage there are also important critiques of attempts to use CHWs to directly replace health workers in achieving UHC, including the potential for poor quality of care and lack of sustainability[9]. Through their community-embedded roles, CHWs have the potential to *link* communities and health systems, for example by conducting health promotion, education and referral to health services. If services provided by CHWs are effective, their location and origin may extend the reach of facility-based health systems to communities or groups who are underserved, poorest, most socially disadvantaged and marginalised. Limited published studies report that CHWs have been critical in supporting equity in the delivery of health services to children, the vulnerable and the poor in marginalised communities[10-12] but the evidence to date is weak and more research is needed in this area[13]. Other types of CTC providers, such as informal private providers may also have important roles to play in creating better linkages to formal health services; for example the Triage and Triage Plus studies in Malawi and Sudan are investigating the potential roles of informal private providers in linking clients to formal TB diagnostic services[14].

Specific social and economic stratifications and norms intersect with particular health service structures in different contexts in variable ways as discussed in the examples from maternal and newborn health, lung health and NTDs below. CTC services may not necessarily overcome all the barriers to accessing services (e.g. direct and indirect costs), but they can be an important and effective component of wider health system strategies to improve access and coverage. There have been recent systematic reviews of the effectiveness of CTC providers in improving health outcomes and factors influencing this[15,16]. However, there is less systematically evaluated evidence available on how these outcomes are achieved (i.e. which specific roles played by CHWs have contributed most to effectiveness) or whether CTC providers are able to reach the most vulnerable and marginalised groups.

Below we give a brief overview of the effectiveness of CHWs in service provision and what is known about their ability to extend services to marginalised groups in relation to maternal and newborn health, lung health and NTDs:

### Maternal and Newborn Health (MNH)

**Effectiveness of CTC providers**: CHWs have undertaken a range of maternal health service provision roles in recent years, including: supporting and promoting utilisation of health services, with a focus on delivery care; and facilitating women's groups using Participatory Learning and Action to improve birth-preparedness. There has been mixed evidence from systematic reviews of the effect of CHW activities on maternal health outcomes. There is some evidence that community based intervention packages and specific service delivery interventions by CHWs (such as the provision of continuous support for women during labour in the presence of a skilled birth attendant and administration of misoprostol to prevent post-partum haemorrhage (PPH)) reduce maternal morbidity[17,18]. There is also evidence that CTC interventions for intra-partum and newborn-care preparedness, specifically those based on building community support groups, community mobilization activities and home visits by community-based workers are effective in reducing neonatal deaths[17,19].

### Equity: reaching vulnerable and marginalised groups

Current research demonstrates heterogeneity regarding the equity impact of CTC providers. In a comparison of the magnitude of inequalities for maternal and child health indicators across 54 countries, skilled delivery was the most inequitably utilised service. Despite having introduced CHW programmes, Ethiopia ranked as one of the most inequitable of 54 countries, and Malawi was the third most equitable and Bangladesh, discussed below, ranked 13th[20]. There is evidence of inequities in access to CTC providers themselves including people living in more remote places,[21] people with disabilities, drug users,[22] people from lower castes[23] and from poorer wealth quintiles[24] receiving less adequate CTC services compared with others in specific contexts.[25].

Some evidence from Bangladesh indicates that CTC services for maternal and newborn health improve equity. For example, community interventions for maternal health care, including an intervention on improving maternal, newborn and child survival using CTC providers such as the female CHWs deployed by BRAC (Shasthaya Shebika, Shasthya Kormi and Newborn Health Workers) to create demand and provide services at community level contributed to more equitable utilisation of maternal health services[26]. These included reductions in the relative concentration indexes according to wealth quintile for antenatal care (ANC), skilled birth attendance and use of modern family planning methods in 2011 as compared with 1993/1994 DHS surveys[25].

However, equity impacts can be complex and multi-directional. A recent evaluation of an intervention which created demand and provided services at community level revealed that, although utilisation of some maternal and newborn health services (including use of ANC care, use of skilled attendants for home delivery and post natal services) became pro-poor over the course of the intervention (as measured by concentration indices), there was no change in equity of delivery at health facility level between income groups, and high income groups became more likely to use of a medically trained provider for ANC over the course of the intervention.[26]. Bangladesh has made considerable gains towards improving equity in child survival. However, despite overall improvement in equity for skilled birth attendance (SBA), with a reduction of the relative concentration index from 0.66 (1993/4) to 0.56 (2011). Large coverage gaps still persist between the wealthiest and poorest groups, with SBA having increased from 3.1% in 1993/4 to 31.0% for quintile 1, meanwhile for quintile 5 SBA rocketed from 11.5% to 63.7%.

### Lung health

**Effectiveness of CTC providers:** Within the field of lung health, Tuberculosis (TB) control is one of many areas in which CHW/lay health workers have been recognized as making a valuable contribution. To date this contribution has primarily focused on direct observation of treatment[27]. Involvement of CHWs and other community members to facilitate DOTS can substantially increase treatment completion rates and reduce client and societal costs, relative to facility-based services. Most studies available to date indicate that “interventions implemented by CHWs are highly cost-effective by international standards ”[28].

**Equity: Reaching vulnerable and marginalised groups:** The role of CTC providers in supporting universal health coverage and extending the reach of intensified case finding for TB amongst marginalised groups is less well documented. Studies report that through standard approaches to TB care and treatment (which rely on passive case finding) men are more frequently diagnosed as having TB[29]. There are a range of possible explanatory factors, including that in some contexts women may experience greater barriers to accessing diagnostic services. The TB REACH project Ethiopia demonstrated the effectiveness of partnership with female health extension workers (HEWs) (who collect sputum at community level at the household or the health post and liaise with supervisors for laboratory follow up): this brought the male:female ratio among sputum smear positive pulmonary TB (PTB+) cases to close to 1:1 and more women were diagnosed with TB at community level than in the health facilities[29]. The proportions of children and elderly cases also increased, demonstrating that CTC approaches can increase access to diagnosis and treatment amongst women, children and elderly[29,30].

### Neglected tropical diseases

**Effectiveness of CTC providers:** The African Programme for Onchocerciasis control (APOC) was launched in 1995 and was the first NTD programme to adopt a ‘community directed’ approach to treatment with ivermectin (CDTI)[31,32]. In the CDTI local community volunteers (often referred to as Community Drug Distributors (CDDs)) have distributed ivermectin once a year to the entire at risk population for up to fifteen years. By 2011, 80.2 million people were receiving treatment[33]. Drawing on the design of APOC the Global Program to Eliminate Lymphatic Filariasis (GPELF) also uses CDDs to deliver preventative chemotherapy to the entire at risk population. Between 2000-2009, more than 2.8 billion doses of medicine were delivered to a cumulative targeted population of 845 million people[34]. Research has demonstrated that the approach is sustainable and effective[31-33].

**Equity: Reaching vulnerable and marginalised groups** There is however limited knowledge about how equitable the coverage is within populations; that is, which individuals/groups have been reached and why. Further, it may be useful to explore what ‘community directed’ means in the context of specific programmes and the extent to which communities are involved, made aware and empowered. CDDs are drawn from their own communities and these are often in remote and hard to reach areas (for example the APOCs programme has delivered drugs to communities, which are more than 20km from any health facility). There are however evidence gaps in understanding the extent to which their knowledge of both context and community is drawn upon to design strategies to maximise coverage. CDDs could be and often are used to deliver other health and development activities[35,36], including malaria treatment, polio immunisation, guinea worm eradication and water protection[36-38].

In summary there is some evidence about the effectiveness of CTC providers in service delivery within maternal and newborn health, lung health and NTDs. This evidence however has reported mixed results in different contexts and different aspects of care.

There is less clear evidence on the extent to which CTC providers are able to contribute to advancing UHC to meet the needs of the poorest and underserved individuals and households; although there are clearly some promising examples of good practice. Further research and programmatic experience are required to assess the extent to which CTC providers can extend the reach of the health system and deliver high quality and effective services to vulnerable and marginalised groups in different contexts. These would require methods to assess effectiveness, quality and equity. In turn equity analysis requires understanding different aspects of vulnerability and marginalisation, for example how the interplay between gender, ethnicity, dis/ability, caste and poverty shape vulnerability of individuals, households and communities in different contexts and with respect to different health issues[39].

## 2. CTC providers as agents of social change: gendered intra household dynamics and links within and beyond the health systems

Gender analysts in health have produced empirical work in multiple contexts to understand decision making dynamics within households. CTC providers as embedded members of their communities have the potential to play a critical role in addressing underlying social determinants of health. Gender and power relations shape vulnerability to ill-health and decisions around if, when and where to seek care; access to quality preventive and curative services and experience of the impact of ill health[40-42]. The following boxes give a brief overview of evidence for these dynamics with relation to MNH, lung health and NTDs:

**MNH:** Gender divisions of labour, norms and identities, access to and control over resources, and limited autonomy and bargaining positions within the family and community limit women's ability to use health-care services including during pregnancy and delivery[43]. This in turn determines women's opportunities to use preventive and curative services during pregnancy, delivery, and the postnatal period. Poor women often do not have access to adequate transportation to health facilities or the cash to pay for it. They may have to negotiate for transportation with men, other family members, or elders in the community, which can cause life-threatening delays in emergencies. Absolute and relative poverty can pose a serious barrier to women's demand for and access to health care. Relative poverty, low education and lower levels of autonomy and decision-making power are associated with lower use of Skilled Birth Attendants in many countries[44]. CTC providers may be strategically placed to understand the challenges women face in accessing care and how this relates to broader societal and infrastructural challenges including gender norms, access to cash and transport[45]. Gender roles and relations also contribute to shaping social determinants of maternal and newborn health outcomes such as long- and short-term under-nutrition, high fertility, Intimate Partner Violence (IPV), and unsafe abortion. Changing these deep-rooted determinants requires action for social change beyond the health system[46-48].

**The text above is adapted from**[49]

**Lung Health:** Vulnerability to developing lung diseases such as Chronic Obstructive Pulmonary Disease (COPD), asthma, and TB is shaped by intersecting social determinants, including poverty, gender and age. For example, household air pollution (HAP) has in recent years been identified as a major risk factor for COPD and as an important trigger for asthma[50]. HAP is strongly associated with poverty, due to lack of access to efficient energy sources, and disproportionately affects women due to their gendered roles, and children, who spend time indoors with their mothers. Other risk factors, including smoking, occupational exposure and traffic pollution may disproportionately affect men in some contexts; for example, due to social norms promoting smoking amongst men and their greater mobility and employment opportunities than women. Poor and less educated men are likely to have greater exposure and less autonomy to avoid risk than their wealthier and better educated counterparts. Prevention of poor Lung Health therefore requires action at different levels from the global to the individual, and needs to include consideration of the social norms and power relations shaping individual capacities and opportunities to avoid risk.

Access to and utilisation of diagnosis and treatment for a range of lung diseases, presenting with chronic cough, is also influenced by intra-household dynamics and social position. Numerous studies globally have identified associations between poverty, gender and delays in seeking and achieving a TB diagnosis[51-54]. Chronic respiratory symptoms are often associated with stigma in many contexts, with differential impacts by gender, poverty and social marginalisation[55][see CAHRD paper LH Cough]. Effective treatment and/or management of chronic lung diseases require prolonged engagement with health services, with associated visit costs for individuals and their families[55][see CAHRD paper LH Costs and LH Cough]. Perceptions and experiences of stigma, conceptualisations of illness, control over time and resources and family and community support also interact to enable or inhibit successful treatment. Health service provision needs to engage with these social and economic barriers and enablers to diagnosis and effective management of illness.

**NTDs:** Neglected tropical diseases can often cause disability. Studies have shown important gendered dimensions of NTDs; for example women with lymphatic filariasis (LF) in the Dominican Republic experience more social exclusion and shame than men[56,57]. In coastal Ghana, men living with hydrocele (accumulation of fluid in the scrotum) were found to experience challenges with work performance, sexual functioning and every day social interaction and relationships[58]. The nexus between poverty and disability is well established in the international literature[59]. Yet, global programmes for control and elimination of NTDs have focused more on the prevention of transmission than on the prevention and management of disabilities. Affected individuals and households are likely to require support and care even after elimination of NTDs. Addressing important knowledge gaps regarding how disability is experienced and who provides care and social support to people living with disability caused by NTDs is required. There are likely to be important gendered dimensions to this[60] as caring for sick relatives often falls on women and girls, with implications for their own health, financial and social capital[61].

Understanding health as part of sustainable development shifts the emphasis of public health strategy towards the social determinants of health[3]. This challenges the focus of health systems, which have historically been oriented towards biomedical prevention and treatment, and requires more attention to social and structural change and inter-sectoral collaboration at every level of society. Addressing NTDs for example requires links with the Ministry of Education (where Mass Drug Administration is carried out in schools) and with actors working at different levels (from the district to the community) in water and sanitation. CTC providers have the potential to play a critical role in such a shift, but this in turn is likely to require rethinking of their training and mandate.

### CHWs as change agents within communities

Some bodies of literature conceptualise CHWs as social change agents, functioning as social and cultural intermediaries at the interface between the health system and the community[62-67]. As change agents, they are arguably strategically placed to facilitate community participation, stimulate critical thinking and act as a catalyst to social action to address the social and cultural determinants poor health status. At the micro-level, CHWs/CTC providers are in a unique position to observe and understand many of the socio-cultural and gender factors that influence health and healthcare use within households and communities[68]. This is due to their socio-cultural embeddedness and frequent contact with individuals in their household and community settings, as compared with relatively infrequent and brief consultations in health facilities away from their social context. This positioning is recognised in some international literature, and in some national CHW policy and strategies. For example in India, Accredited Social Health Activists (ASHA), primarily linking to maternal and newborn health services, are expected to play the role of a ‘social change agent’[69] as described in their guidelines: “*ASHA will be a health activist in the community who will create awareness on health and its social determinants and mobilize the community towards local health planning and increased utilization and accountability of the existing health services*”[70], although they face challenges in realising this role[63].

In a recent review of the evidence of how context influences the performance of CHWs, gender arose as an important factor[71]. Field experiences from the REACHOUT project suggest that CHWs have to negotiate gender and power relationships within households and communities in their routine work. They often come across social determinants of health, including food insecurity, Intimate Partner Violence and alcohol abuse, and may not feel able to address them. However, there is relatively little published knowledge about how CHWs view these social determinants of health and how they attempt (or not) to deal with them in their interactions with individual clients, the community and the health system. Effective action to address social determinants requires critical reflection on how societal structures and power relations influence individual health outcomes. As members of the communities they serve, CHWs are likely to have internalised the very social and cultural influences that they will need to tackle at individual and community levels. Realising the potential of CHWs therefore requires a process of awareness-raising for providers themselves, which is rarely offered within formal health systems. Other CTC actors outside the formal health system, such as women's group leaders or human rights activists may have more critical perspectives, but are rarely effectively linked to formal health services and may even be seen as oppositional to them.

The potential influence of CHWs may also be limited due to their positioning within gender and other power dynamics in their community. Evidence from Pakistan reveals how Lady Health Workers must operate within the same gender systems that necessitate their appointment in the first place. The interplay of gender, class and hierarchy means that, like the women they serve, they are also marginalized and disadvantaged by the male-dominated context[72]. However, there is some evidence that working as a CHW may contribute towards a process of empowerment for women, who are challenging social constraints and stereotypes in many contexts by having regular employment; being mobile within communities; and fulfilling a socially valued role[73]. Explicit training on critically analysing the social determinants of health has the potential to place such personal experience within a wider frame of understanding, which points to the need for collective action.

In addition, skills development is required to enable CHWs to facilitate a similar process of critical reflection in their communities. Such a process of reflection requires work with peer groups, as well as with individuals and households in their communities. There are examples of participatory community-based interventions in health, such as the Stepping Stones training manual[74], which aim to address a range of inter-related social determinants; however they require skilled facilitation. Training of Community Health Workers in facilitating participatory processes would contribute towards enabling them to play a role as catalysts of social change. A recent systematic review found that participatory learning and action with women's groups, often facilitated by local women with brief training is cost effective in reducing both maternal and neonatal mortality[75]. CHWs could be potential facilitators of such interventions with appropriate training and a mandate to work with community groups. However further consideration is needed of how they may play a complementary role to grassroots development organisations as well as the activities of other governmental sectors.

### CHW roles in strengthening information flows and accountability

We have argued that CHWs have privileged insights into the social determinants of health in communities that may be sharpened through increasing their critical awareness. In addition to direct action to address these determinants there is a need for these insights to inform policy for both health and inter-sectoral policies and priorities, for example by sharing the concerns of communities on quality and nature of services offered[76]. However, there is limited knowledge on the extent to which CHWs are given opportunities to feed into health systems priority setting and bring their embedded knowledge to health systems debates. Mobile technologies have potential to enable CHWs to collect, analyse and use information from their communities as well as access information remotely[77,78]. This arguably has transformational potential to support CHW's stronger participation in generating data, feeding into the health system and informing decision making processes. However, capacity building is required to enable the appropriate analysis and understanding of the data generated, both within health systems and importantly within communities themselves. The utility of information depends on improving the responsiveness of the health system to take action on the basis of data and feedback from communities.

The hierarchical nature of health systems may often serve as a barrier to responsiveness to information from CHWs in their position as the least powerful actors operating at the periphery or lowest level of the system. The development of explicit channels for information flows and mechanisms for promoting the use of information in decision making processes is therefore required to realise this potential. Capacity building of CHWs to facilitate community groups to understand and make use of information about their own health needs and rights has the potential to contribute towards strengthening the accountability of health services to communities. Capacity building on using data for change (through for example quality improvement cycles) can support change. However, deeper understanding is needed of how far CTC providers are accountable to their communities and the structural enablers of such accountability. For example, little is known about the range of approaches used in CTC provider recruitment or selection, including the extent to which they may be expected to serve local elites or the interests of the health system rather than those who are less powerful in communities.

Further research is therefore required to understand the opportunities for the reorientation of CTC provider roles, training and mandate to identify and address the gendered social determinants of health at community level, and to support inter-sectoral collaboration on health. Implementation research is needed to assess how health system decision making processes and structures can be better organised to enable CTC providers to inform priority setting and to complete feedback loops to communities. Finally, better understanding is needed of the potential of CHWs to support the promotion of health service accountability to communities, including the role of facilitating community use of information.

## 3. Retaining, motivating and supporting CTC providers to realise their potential: the need for a health systems and human resources for health approach

We have argued that CTC providers may be strategically placed to support both the delivery of universal health coverage (theme 1); and to use their knowledge to inform health sector development and cross sectoral working to address the social determinants of health (theme 2). These are both challenging roles to undertake however and there is need to be realistic about what is appropriate in different contexts, and develop strategies to support CTC providers to realise their potential and to contribute effectively to health programmes. From a human resource management perspective, challenges include recruitment, turnover and performance. Good practices for managing and supporting CTC providers can be identified and replicated with modifications appropriate to the context. Programmes making improved linkages with informal CTC providers such as informal private providers or TBAs face additional challenges of the general lack of accountability of these providers to the formal system. Recent theoretical debate and analysis has outlined the importance of supporting CTC providers through a robust Human Resource for Health Plan and that this is turn can better support their linkage with the health system[79]. Here we outline 2 key areas of management of CTC providers that are particularly relevant to the issues of equity in coverage and social change: (1) incentives and remuneration: how to attract and retain CTC providers; and (2) performance, workload management and supervision.

### Incentives, remuneration and sustainability

This is a key area of debate[80]: some CTC providers are paid a regular salary and are seen as part of the formal health system (e.g. Health Surveillance Assistants in Malawi); others are paid incentives in relation to performance e.g. numbers of women referred/supported (e.g. *Shastaya Shabikas* in Bangladesh) and others are “volunteers” in the true sense although they may receive allowances for transport or meeting attendance or are helped by the community in agricultural activities (e.g. CDDs for NTDs). The management strategies for attracting, retaining and supporting the performance of people working on a voluntary basis or without formal contract are more delicate as intrinsic motivation is likely to have greater importance. There is therefore a need to understand the reasoning for the selection of a particular CTC remuneration model and what motivates CTC providers - particularly volunteers – in different contexts and to identify the challenges and opportunities CTC providers face in their work. CTC volunteer providers are often women from poor rural communities, who may be motivated by factors including improved community status, career opportunities, and altruism. This is likely to vary according to context, by gender and by community, and there is evidence that programmes can fail to attract the (female/male) workers they require, with a recent study in Kenya finding that CTC provider roles (community health worker and community health extension worker) most frequently attracted women, while other roles, such as pharmacy attracted men[81]. Incentives (both intrinsic and extrinsic) are key to performance, sustainability and career path choices and are an area that requires further research. Looking critically at how best to support and enable CTC providers is arguably an important opportunity to contribute towards women's empowerment.

### Performance, workload management and supervision

Effective management of CTC providers requires clarity on the optimal package that specific providers can deliver in a given context, training, resources and supportive supervision, Ineffective planning, management and supervision of CTC providers as human resources for health may contribute to high staff attrition, and poor quality and effectiveness of services. In many contexts supervision focuses on reaching targets rather than empowering and supporting community health worker needs. Meanwhile in Mozambique supervision was at times described as fault finding rather than supportive. This had negative consequences for openness during supervision with CTC providers tending to hide gaps during subsequent supervision visits[82]. Supervision structures and processes rarely focus on the gendered experiences of CTC providers themselves or how social norms affect their work and interaction with communities. Supervision and support is further complicated from a health systems perspective by poor co-ordination between vertical, health topic-specific programmes that use CTC providers for service delivery, and limited consideration of the multiple workloads and competing priorities that CTCs face. CTC services often lack monitoring and evaluation (M&E) systems and referral mechanisms to formal health facilities are poorly tracked or recorded. In summary, to realise their potential, strategies are needed to motivate, strengthen and support CTC providers and co-ordinate between the programmes giving them tasks.

In summary we argue that CTC providers have strategic potential to support the Sustainable Development Goals, the equity and responsiveness of health systems and to promote universal health coverage (range and reach of services). Their community links and interface role between communities and health systems means that are also well placed to be a voice for community priorities. Realising this potential requires strategic, supportive and sustainable investment and developing the evidence base in the following areas:

## Questions for research in the 10-20 year horizon

1. What are the best approaches to assessing the extent to which CTC providers reach and meet the needs of different vulnerable groups, including the interplay between different axes of vulnerability – gender, ethnicity, dis/ability, caste, poverty etc. *(intersectional analysis)*

2. How can health system decision making processes and structures be better organised to enable CTC providers' experience to inform priority setting?

3. What is the potential of mobile technologies in enabling CTC providers to better collate, analyse and act on community health priorities?

4. How can the accountability of CTC providers to their communities be best understood, enabled and monitored?

5. What are the opportunities and challenges for CTC providers to support health systems and to better address the gendered social determinants of health at community level?

6. How can CTC provider's insights feed into and support inter-sectoral collaboration with different sectors (e.g. education, transport, livelihoods)?

7. What are the best approaches to motivate, retain and support different types of female and male CTC providers in specific contexts? Does capacity development amongst CTC providers contribute towards social change?

## Competing interests

No competing interests

## Authors contributions

ST and RT conceptualised and drafted the paper. EM produced the Neglected Tropical Diseases examples. RM provided additional case study examples. All authors contributed to points in the framing and analysis of the paper and read and approved the final version. ST, RM and RT part of the Reeachout Consortium and are grateful for inputs and comments from Reachout Consortium colleagues.

## Declarations

This article has been published as part of *BMC Proceedings* Volume 9 Supplement 10, 2015: The 2014 CAHRD (Collaboration for Applied Health Research & Delivery) Consultation. The full contents of the supplement are available online at http://www.biomedcentral.com/bmcproc/supplements/9/S10.
